# Men’s views on causes and consequences of erectile dysfunction or premature ejaculation in a primary care population: a qualitative study

**DOI:** 10.1080/02813432.2024.2327501

**Published:** 2024-03-31

**Authors:** Elin Gahm, Magnus Peterson, Kjerstin Larsson

**Affiliations:** a Department of Public Health and Caring Sciences, Section of Family Medicine and Preventive Medicine, Uppsala University, Uppsala, Sweden; b Academic Primary Health Care, Region Uppsala, Sweden; c Department of Public Health and Caring Sciences, Section of Health Equity and Working Life, Uppsala University, Uppsala, Sweden

**Keywords:** Erectile dysfunction, premature ejaculation, primary health care, sexual dysfunction, qualitative research

## Abstract

**Objective:**

To explore men’s views on the causes and consequences of two common sexual dysfunctions – erectile dysfunction and premature ejaculation – and how this affects physical and mental health as well as social life and intimate or close relations.

**Design:**

A qualitative design with semi-structured interviews using open-ended questions was employed. Individual interviews were conducted, audio recorded and transcribed, and a qualitative content analysis of the text was performed.

**Setting:**

Informants were recruited from an outpatient primary care clinic in Sweden that offers consultation about sexual health to primarily younger men, age 20 years and above.

**Subjects:**

A total of 18 participants were included in the study, ten with erectile dysfunction and eight with premature ejaculation or both.

**Main outcome measures:**

Using the content analysis, different views and strategies of erectile dysfunction and premature ejaculation were presented to illustrate a range of perceptions.

**Results:**

The main theme emerged as ‘Striving to understand and deal with the problem’, which was divided into four categories: ‘Reasons for seeking healthcare’, ‘Own perceptions/images about the problem and its cause’, ‘Experienced consequences on sex life’ and ‘Relationship qualities’.

Participants experienced their problems in relation to a partner. Feelings of shame and fear of not being fit for desired sexual practices were common. They thought that underlying physical illness or previous sexual activities could have caused their problems. Decreased sexual desire and low self-esteem were seen as consequences, and participants wished for both medical treatment and counselling as support.

**Conclusion:**

Sexual dysfunction impairs general health and relationships with partners. While counselling is the basic treatment, those who are offered pharmaceutical treatment need follow-up concerning effectiveness and potential concerns.

## KEY POINTS

- People with sexual dysfunction may have decreased quality of life and poorer somatic and mental health in general.
- People with sexual dysfunction may have few places to turn to in a traditional primary health care setting.
- The participants expressed concerns about their physical health, their relationships with partners and motivation for treatment.
- Men’s visits to primary care for sexual dysfunction provides opportunities to talk about lifestyle and relationships and discover other illnesses.

## Introduction

Sexual dysfunction among men is a highly prevalent, multifactorial condition, often associated with both common chronic somatic and psychiatric conditions. [1–6] As such, it requires assessment from a biopsychosocial perspective, which can ideally be performed by general practitioners who typically see large numbers of unselected patients and also have experience and ability to address complex conditions. [3–5,7,8] However, to sufficiently address the issue, it is important to understand better the patients’ perceptions of how the sexual dysfunctions impact their physical and mental health and sociocultural situation.

Erectile dysfunction (ED) and premature ejaculation (PE) have reported prevalence from 7 to 17 and 10 to 15%, respectively, of men in Sweden [1], Great Britain [9] and Denmark [10]. These numbers are self-reported and thus defined by the respondents and captured in studies among the general population.

ED and PE often coexist, although the potential common pathophysiological pathways and connections are not clearly established. While ED is associated with other conditions, such as hypertension and type 2 diabetes mellitus, and age, this is not observed for PE. Possible linking factors between ED and PE, which have yet to be clarified, include ED as a consequence from attempts to reduce sexual excitement due to risk of PE. If a man with ED requires intense stimulation to maintain erection, it could potentially lead to PE and when PE results in early detumescence it may present as ED. [2] Due to the controversy in diagnostic criteria and the limited use of standardised questionnaires on this issue, the link between PE and ED still needs further clarification. [9]

Experts express that all men seeking professional help for sexual dysfunction should receive basic psychosexual education. [11] Moreover, the World Health Organisation (WHO) is clear about the need for health care providers to be able to deal with problems related to sexuality. [12] The government’s official investigations in Sweden have consequently highlighted the need for health care providers to improve the assessment and queioning regarding men’s health. [13] This is important as it both relates to physical and mental health and is also essential to changing the stereotypical pattern that men are less prone than women to seek health care. [3,13,14]

A Norwegian study showed that 47 out of 1,117 (4.2%) general practitioner (GP) consultations dealt with sexual problems. Of these consultations, 19 were with men, with most of them having concerns about ED. [7] There is a lack of knowledge on challenges faced by GPs in addressing sexual problems in consultations with patients, but lack of experience with sexual health issues is one barrier for bringing it up. [8] The available literature indicate that sexual and reproductive health is a domain for women and not sufficiently prioritised for men. Lack of knowledge and no clear entry for men into this domain indicate the need for improvements in the medical education and in health system interventions. [15] The proportion of males who discuss PE with their GP can be greatly increased by even a simple measure such as training GP:s in communication strategies on this subject. [16]

Based on present knowledge that ED and PE are commonly associated with other diagnoses handled in primary health care, it is important to investigate this further in a primary care population. Especially as it is also known that ED and PE can lead to significant emotional distress, with difficulties in addressing the topic, both among patients and health care workers. [4]

ED is classified into primary organic or primary psychogenic, though most cases are of mixed aetiology. The pathophysiology may be vasculogenic, neurogenic, anatomical, hormonal, drug-induced and/or psychogenic.

The aetiology of PE is unknown with few data to support biological and psychological hypotheses. It is hypothesised that lifelong PE is mediated by a complex interplay of central and peripheral serotonergic, dopaminergic, oxytocinergic, endocrinological, genetic and epigenetic factors. Acquired PE may occur due to psychological problems and/or co-morbidity, including ED, prostatitis and hyperthyroidism. [17] The wish for providing sexual satisfaction to a partner may be yet another (social) cause of premature ejaculation. [18]

Due to the scarcity of research in this area it is justifiable to research this field of interest using a qualitative rather than quantitative study.

## Aim

This study aims to explore men’s views on the causes and consequences of their sexual problems, specifically ED and PE, and how they affect physical and mental health as well as social life and intimate or close relations.

## Material and methods

### Design

In order to investigate feelings, thoughts, experiences and coping with problems related to PE and ED, a qualitative research study using content analysis was conducted. [19] The reporting was carried out according to the Consolidated Criteria for Reporting Qualitative Research (COREQ), a well-established checklist for qualitative studies. [20]

### Participants

The study was conducted at an outpatient clinic that offers consultation and care in sexual health to men aged 20 years and above; the majority of visitors are 20 to 29 years old. Potential participants were informed about the study through posters at the clinic’s reception and through the clinic’s nurse. A purposeful sampling was used and the participants were consecutively recruited among the men who met the inclusion criteria: seeking help for ED and/or PE and who spoke Swedish or English. Written informed consent was obtained from those who chose to participate. A total of 18 men agreed to participate in the study, including ten with ED and eight with PE or both. Six patients with ED and three with PE were given verbal information about the study and the possibility to participate, but declined participation. All of the consenting participants went through with the interview. See Table 1 for participants’ characteristics.

**Table 1.** Participants’ characteristics.

No	ED/PE^a^	L/A^b^	Age	Other comorbidities and medications	Steady partner^c^	Occupation
1	ED		25	Herpes simplex in eye/Valaciclovir	Yes	Student
2	ED		22	Acne/Isotretionin	Yes	Student
3	ED		21	0	Yes	Student, employed
4	ED		21	0	Yes	Student, employed
5	PE	A	31	Benign vertigo, back pain. Previous depression	Yes	Employed
6	PE	A	30	ED, ADHD	No	Employed
7	ED		26	0	No	Student, employed
8	ED		24	Previous Tick Born Encephalitis and pneumonia	No	Student
9	ED		30	0	Yes	Employed
10	PE	A	23	ED, gastric hernia/Omeprazole or Gaviscon	Yes	Student, employed
11	ED		20	0	No	Student
12	PE	L	28	ED	No	Employed
13	ED		23	0	No	Student
14	ED		28	Migraine rarely Allergy/Antihistamine	Yes	Employed
15	PE	L	27	ADHD	Yes	Student, employed
16	PE	L	65	Cardiovascular disease/Metoprolol, Acetylsalicylic acid, Rosuvastatin, Ezetimibe, Candesartan	Yes	Retired
17	PE	L	41	0	Yes	Employed
18	PE	L	22	0	Yes	0

^a^ ED/PE: Erectile dysfunction/Premature ejaculation.

^b^ L/A : Lifelong/Acquired.^c)^One single partner only.

### Data collection

**Figure 1.**
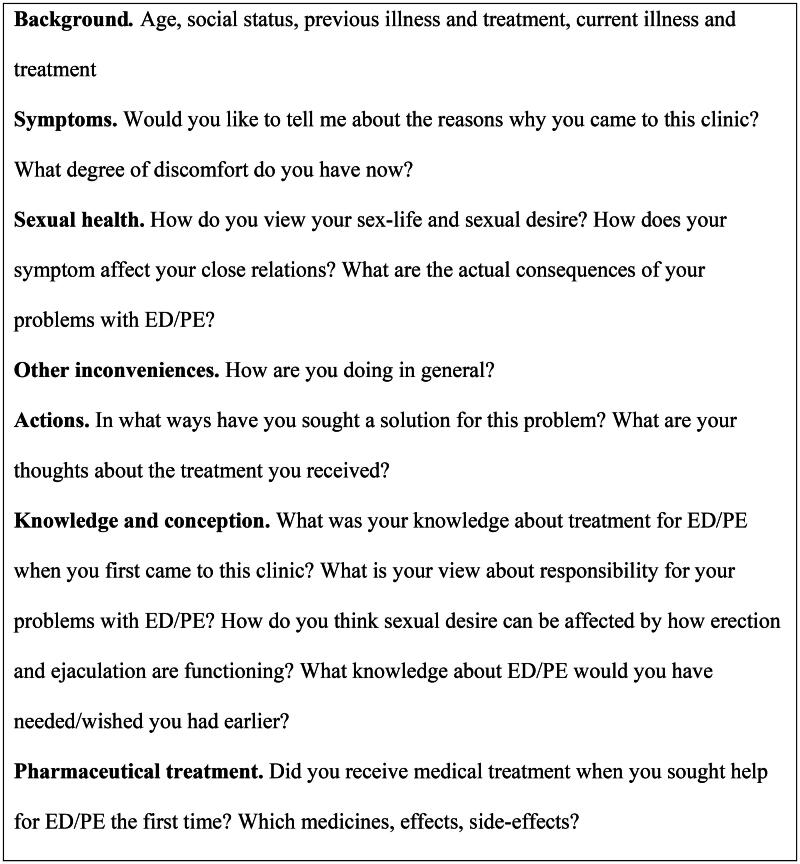
Interview guide.

The interviews were performed using a semi-structured interview guide (Figure 1). All participants were asked the same questions in the same sequence with some difference in supplementary questions, depending on the participants’ answers. All interviews were conducted by phone because of the restrictions regarding face-to-face meetings due to the Covid-19 pandemic. When no new information was forthcoming from the last two or three interviews, the sampling was terminated.

**Figure 2.**
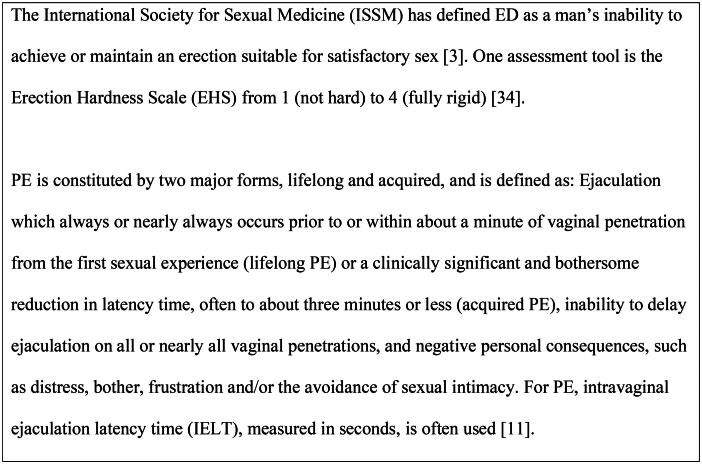
Diagnostic tools [3,11,34].

The duration of the interview was 45 min on average. The interviews were audio-recorded and transcribed, comprising a total of 217 pages. Notes were not taken during the interview, and no one was interviewed more than once.

### Data analysis

A qualitative content analysis was performed as follows. [21] The first five transcribed interviews were read word by word, and meaning units, i.e. words and sentences in the text that reflected the purpose of the study, were identified by the first author (EG) and the last author (KL) separately. The first author condensed the meaning units. Then, the first and last author coded the meaning units from the first five transcribed interviews together, compared the codes for similarities and differences, and grouped them into a preliminary structure of subcategories and categories.

The following interviews were coded by the first author, who also suggested subcategories and categories that were continuously discussed with the last author. The subcategories and categories, organised into a central theme derived from the data, were discussed among all authors until an agreement was reached about the structure. [21] No computer software was used for analysis and data storage.

## Research team

The interviewer (EG) is an experienced general practitioner, educated in sexual medicine, working at the clinic from which the participants were recruited. She is a medical doctor with an academic interest in the field and a record of previous independent publications, among others as editor of a text book on sexual health. The second author (MP) is a general practitioner specialised in pain treatment, who meets patients with long-term pain that face these problems. The last author (KL) is a medical social worker and qualitative researcher. Both MP and KL are PhDs.

The study was co-funded by a pharmaceutical company developing treatment options for sexual dysfunction, which influenced the choice of topic for the study. However, the company had no influence on the conduction of the study, and neither the analysis of the data nor the preparation of the manuscript.

## Contact with the participants

Some of the participants had previously consulted the interviewer before the interview took place. Only the interviewer and the participant took part in the interviews.

### Ethical considerations

The participants received both oral and written information about the study, and that their potential participation was voluntary, that confidentiality was guaranteed and that they could decline to answer any questions or withdraw from the study at any time. Any urgent medical issues were prioritised over participation in the study. Those who had not yet received counselling were treated in connection with their participation in the study. This study was approved by the Ethical Review Authority in Stockholm (Dnr 2020-00596).

## Results

### Striving to understand and deal with the problem

The results encompass a main theme, namely striving to understand and deal with the problem. Central to the participants’ narratives was their desire to seek help to increase their understanding of the problem, and their reflections about experiences of managing and coping in various ways with erectile dysfunction and premature ejaculation. Four categories with related subcategories emerged from the analysis for both ED and PE: 1) Reasons for seeking healthcare, 2) Own perceptions/images about the problem and its cause, 3) Experienced consequences on sex life, and 4) Relationship qualities. See Table 2 for an overview of the theme, categories and subcategories.

**Table 2.** Summary of themes, categories and subcategories that emerged in the qualitative analysis of interviews about erectile dysfunction (ED) and premature ejaculation (PE).

Theme	Category	Subcategory
Striving to understand and deal with the problem	Reasons for seeking health care	Exclusion of physical illness Seeking help with medication or counselling
	Own perceptions/images about the problem and its cause	Feelings of embarrassment and inadequacy Changing lifestyle factors (only for ED) Pornography/sex restrictions (only for ED)
	Experienced consequences on sex life	Avoidant behaviour Effects of sexual desire Specific sexual activities (only for PE)
	Relationship qualities	Saving important relations Relationship significance

#### Reasons for seeking healthcare

*Exclusion of physical illness.* The reason for seeking professional help was often to exclude physical illness as a cause of the problem. Many were aware that both ED and PE could be caused by mental stress as well as hormonal deficiencies and believed they needed a physical evaluation to move on. Another concern was related to fertility.

But since I have only managed twice [to have satisfying penetrative sex] in all these years, it means… you have to do it in some other way to be able to have a child… That’s another issue. (Participant 13)

##### Seeking help with medication or counselling

Most of the participants came to this specific clinic hoping to get help, without specifying whether it was from medicine or counselling. Others expressed a wish for medical treatment. Participants recounted stories about being disappointed that previous doctors had not prescribed medication but only advised them to practise various non-pharmacological techniques.

Some participants with ED were sceptical about pharmacological treatment regardless of whether or not they had tried it. They argued that needing such treatment at a young age made them feel like a failure, and they were afraid of becoming dependent on medications. The participants who suffered from both ED and PE had negative attitudes, similar to those with ED, including a fear of becoming dependent on the medication.

Positive attitudes towards pharmacological treatment were based on a strong desire to receive help, while ambivalent attitudes included a sense of comfort that there was a possibility to try pharmacological treatment in the future if other remedies turned out to be insufficient. Participants with lifelong PE had a positive attitude towards pharmaceutical treatment.

Some participants had sought help previously. One participant recalled being told by a physician that his problems with PE would disappear once he got married, although the physician was still willing to prescribe SSRI.

#### Own perceptions/images about the problem and its cause

##### Feelings of embarrassment and inadequacy

It was common for the participants to express feelings of embarrassment over their problem with ED or PE. The feelings were most obvious in the sexual situation and in situations where they needed to discuss the problem with their partner. Some participants expressed this directly. Others described feelings of not being sufficient overall, or a fear of not being good enough in the sexual situation.

My brain goes into overdrive, going through all kinds of possible situations; what did I say? When will we see each other next time? Will it be possible to have sex then? (Participant 12)

Participants recounted a sense of failure also in a social context among male friends, leading to an unwillingness to talk about and expose the problem.

##### Changing lifestyle factors (for ED)

A common idea among the participants with ED was that their problems could be due to an unhealthy lifestyle. Most participants knew that good overall health and a healthy way of living can play an important role in erectile function.

I started working out more since I figured it [the ED] could be due to my weight and so I did what I could. I tried to eat better and work out more, quit smoking. (Participant 6)

##### Pornography/sex restrictions (for ED)

Some explained their erectile insufficiency with having had too much sex or watching too much pornography. They figured that the gap between the observed images and reality would make them less aroused in sexual situations with a partner. There was an idea that abstinence from pornography, or from having what was considered too much sex, could calibrate them back to what they believed to be normal.

I thought it was a question of sensitivity, that if I give it [masturbation] up for a longer period, it may be easier when I have sex, but it didn’t quite work; not really effective. (Participant 9)

#### Relationship qualities

##### Saving important relations

Several participants stated that the reason for seeking help at this particular moment, was because the conditions for having sex had changed. For example, their current relationship was more important than previous relationships; the current partner wanted to have sex more often than previous partners did; it was the first time that the participant was in a serious relationship; that the relationship was in jeopardy because of the sexual dysfunction; its effect on overall satisfaction with the relationship.

In addition to experiencing less pleasure themselves, participants believed that the partner may not enjoy the sex as much either and that the dysfunction ruined the joy of sexual activity.

So, this is not fun for me or for her because she also wants to see me enjoying [sex]. (Participant 1)

The participants and partners stated that the expectation of penetrative sex was a limiting factor in the possibility of having satisfying sex. Several participants expressed a direct fear of losing their partner due to lack of sexual performance.

This was the first time I was in a serious relationship, and I feel the sex was quite important, so like… I was more in a hurry to try to solve this, like I didn’t want this to end because of the sex life. (Participant 2)

##### Relationship significance

Support from current and previous partners was important. Moreover, the participants generally believed that communication within the relationship would help them relax, and therefore they would be less troubled by the sexual dysfunction.

When it’s something that mostly happens in a couple’s situation, it’s good to talk about it. I think that’s more helpful than for one partner to just tell the other one to solve it yourself. *(Participant 14)*

The participants discussed their own expectations of sex with their partner, and how these expectations could affect the sexual experience. Expectations were that sex would be less of a problem if practised with a desired partner. The degree of suffering and how the problem affected the sexual satisfaction, were aspects considered as depending on the quality of the relationship.

She cared; it seemed as though she, kind of, was ok. Like, kind of supportive and that made it easier immediately when she did not push me. (Participant 2)

A wish to have sex without any emotional commitment could be one reason to inquire about medication. There was the perception that reality will never live up to one’s imagination. Satisfying sex requires romantic feelings but if one should fall in love, fantasising about sex with that person inevitably collides with the idea of sex in reality.

I guess you should have realistic expectations and not fantasise. You are supposed to have feelings involved [when having sex] and I don’t want that. I just want to do it, just grab and go, and that’s not possible. (Participant 7)

Another finding was that sex could be used as a way to have closeness and intimacy, without a real desire for sexual value.

I sleep around a lot but mostly because I like the closeness, which I actually get, but the sex itself is often a bit of an anti-climax. (Participant 13)

#### Experienced consequences on sex life

##### Avoidant behavior

The stress that the participants experienced from their sexual dysfunction could result in avoidance of sexual situations or situations that might lead to sex. They would sometimes avoid contact with a potential sex partner, despite having a desire to develop a relationship, and they deselected potential partners. Even if it was appealing to have sex with a certain person, it was also important to feel safe in case of failing.

The participants agreed that compensating for penetrative sex with other sexual practices was a way of coping rather than a desirable solution. Giving the partner oral sex or caressing could replace penetrative sex or masturbating, instead of having sex with a partner even though the latter was preferred.

Instead of seeking sex [with the partner], I have masturbated because I can manage that. But it seemed pretty unfair. (Participant 5)

##### Effect on sexual desire

Some participants experienced that the level of desire could become lower at the time of the sexual activity because of the sexual dysfunction. A hint that the erection might dwindle could decrease the desire to a minimal level during ongoing sex. The fear of failure to perform could result in associating the sexual situation with anxiety and decrease the motivation to have sex. Sexual desire and resistance to sex could be present at the same time.

No, it [ED] doesn’t affect my desire; it affects my performance. (Participant 7)

*Specific sexual activities (for PE)*. The ability to control ejaculation could be impaired in specific sexual situations. One participant described that it was easier to control ejaculation when engaging in an activity that involved being submissive, relative to the partner, a role he preferred. Another participant had more difficulties to control ejaculation when engaging in a preferred activity, such as penetrative sex.

It’s more when penetrating because that’s so much… that’s very sexy for me, the penetrative sex. (Participant 12)

## Discussion

The analysis revealed that the main theme was the participants striving to understand and deal with the problem. They sought help to increase their understanding of the problem, and they reflected on their experiences to manage and cope with erectile dysfunction and premature ejaculation in various ways. The main findings were the participants’ experiences of their problems in relation to a partner and their concerns about their own physical health. Feelings of shame and fear of not being fit for desired sexual practices were common. Decreased sexual desire and low self-esteem were viewed as consequences, and participants wished for both medical treatment and counselling as support.

### Findings in relations to other studies

Vik and Brekke showed that the issue of sexual health was discussed in 4.2% of the 1,117 GP consultations. However, their study did not show whether the reasons for the low number was due to a reluctance by the patient or the doctor to address the issue, or if it was dependent on other causes. [7] Other previous studies have described the reluctance to bring up issues about sexual health with a clinician, for reasons such as embarrassing oneself and the doctor. [4,5,18,19,22] Studies on barriers to seeking help have identified, for instance, perceptions that ED is a normal effect of ageing [23] and that the physician would dismiss sexual difficulties as trivial. [24–25] In our study, we asked for motives for actually seeking help, rather than what hinders it. Fear of illness was one important reason, which has previously not received much attention in primary health care.

Another reason for seeking help was perceiving the partners’ frustration with their sexual relationship. A previous study showed that shame and embarrassment are common results of sexual dysfunction, and that ED constitutes a challenge to intimate relationships. [24] Partners of patients suffering from PE have reported avoiding discussing the problems due to fear of embarrassing their partners. [26] Feelings of embarrassment among our participants were further expressed as a fear of not being good enough, or a sense of inadequacy in the sexual situation. The findings in our study are congruent with prior observations that men’s sexual satisfaction is strongly influenced by that of their partner’s. [27]

Previous reports have indicated that one type of sexual problem is often associated with other, which is also supported in the present study, where we observed that a loss of sexual desire was reported as a consequence of ED and PE. [27–28]

Revicki et al. found that female partners reported that their male partners reduced the number of sexual advances due to anxiety related to PE. [26] Avoidance of sex and intimacy with partners, due to ED or PE, may itself cause decreased sexual desire. This was true for our participants, as sex meant a risk of disappointment. However, loss of desire was not the case for all of our participants, and the level may well be unchanged despite ED or PE. The reasons for having sex may be other than sexual desire, for instance, a desire for intimacy and closeness to a partner, and the need for adequate erection and sexual stamina may primarily emanate from the partner. Further, men with a female partner suffering from sexual dysfunction are associated with a four-fold risk of ED and doubled risk of PE. Interplay between partners in an intimate relationship is complex where sexual dysfunction can become a vicious cycle. Clinicians should therefore assess sexual dysfunction with a wide approach. [18]

In a previous study, fear of side effects and anxiety were reasons for rejecting medication. [29] This is in line with our findings, as well as lack of spontaneity and the fear of drug dependence.

Participants with PE were, in general, more positive and eager to try medication than those with ED only. A few had been offered SSRI, and there were accounts of tramadol prolonging time to ejaculation, which has also been previously noted and assumed to be due to the serotonergic effect of tramadol. [30–31]

Another finding was a notion that overusing the sexual organs – e.g. by having either too much sex with a partner, masturbation, or consuming too much pornography – could lead to a dysfunction. Contrary to this belief, clinical sexologists have argued for the use-it-or-lose-it principle [6], and there is little scientific support for theories and perceptions of the detrimental effects from masturbation and consumption of pornography. A possible reason behind this perception from one of the ED participants may be that sex is not always a pleasurable experience. What could drive one to have sex may be something other than desire, possibly anxiety, boredom or eagerness to satisfy a partner.

### Strengths and limitations

Credibility of the result is strengthened by posing the interview questions in an open-ended fashion, and by allowing the data to be processed by researchers with different levels of pre-understanding of the studied topic. The interviewer/first author (EG) and the second author (MP) were familiar with the research field. The interviewer as educated and experienced in sexual medicine, and the second author, as a general practitioner, who meets patients with long-term pain that face these problems. The last author (KL) had no pre-understanding of the studied field, which may minimise the risk of preconceptions in the analysis process. To achieve confirmability, quotations were used to illustrate the findings.

The interviews were conducted over telephone, which may be considered a limitation since a qualitative study is supposed to intercept nonverbal communication as well. However, an advantage of not interviewing face-to-face could be that any unclearness must be illustrated with words and thus might be more explicit.

A limitation of this study is that the sample was consecutively drawn from a single outpatient clinic specialised in sexual health in men. However, the participants in this study are potentially more likely to speak openly and candidly about their experiences than the general population suffering from ED and PE. It is also possible that they are more distressed because of their sexual dysfunction than the general population of men. Almost all (16 out of 18) participants were also younger men age 20—31 in reproductive age, which may have affected the perspective of their experiences. Two participants reported having ADHD, one previous depression, one previous tickborne encephalitis and one (rare) migraine (Table 1) and it cannot be ruled out that these psychiatric and neurological conditions could have affected sexual function, considering the close link to mental health. However, these are common conditions and the results from the study could still be considered transferable to a primary health care population of men in general, based on the fairly wide selection of participants, even though the sample in this study consisted primarily, but not exclusively, of younger men.

The under-reporting of mental and physical symptoms in men, linked to sexual function that emerged in this study, is probably also found in other countries with similar conditions as in Sweden. The results are also likely to be transferable to other European countries, considering the WHO’s finding that the need for men’s reproductive and sexual health are not being met by the health systems in this region. [12]

Hopefully, insights from our participants’ narratives can guide clinicians in primary health care to address issues of sexual health in their encounters with men seeking health care.

### Meaning of the study: possible mechanisms and implications for clinicians or policy makers

What distinguished the participants with ED from the ones with PE in our study, was the perception of good overall physical health as a prerequisite for adequate sexual function. Many had attempted to modify their lifestyle factors before seeking medical consultation.

Participants with PE focused more on the importance of their mental status, specifically in the sexual situation, and experiences emerged of being able to function better when having more sex, defined as having more ejaculations. Moreover, PE made it impossible to engage in a few desired sexual activities, hindering enjoyment from sex. In our study, the problem of controlling ejaculation was often related to certain sexual practices, such as penetrative sex.

When a man with sexual dysfunction seeks primary health care, it is an opportunity to get information about his health in general. Health issues are often seen as a question for women rather than men. However, it is important for people working in primary health care to be aware of men’s actual concerns, about their own lifestyle and relationships, when bringing up issues of sexual dysfunction.

In previous research on the process for diagnosing prostate cancer, the importance of flexible and tailored information that meets the needs of the individual has been discussed. [32–33] Even though these studies concern prostate cancer, and the study participants were significantly older than our participants, it may still be an issue that needs to be addressed in GP consultants about sexual health.

Our findings support that clinical assessment of men seeking primary health care because of sexual dysfunction, can be facilitated if they are asked questions about how it affects their life in general and if relationship issues are addressed. This can contribute to reducing relationship problems and beliefs about sexual function, and to improved self-esteem. For example, by asking open-ended questions such as ‘How does this affect your life in general and also, your intimate relationships?’

Further, querying about the knowledge regarding available medical treatment, as well as offering counselling, can improve the quality of the consultation.

When evaluating the health status of men who are struggling with their well-being and seeking health care, it may be helpful to ask about their sexual health. The results from this study can contribute to making primary health care workers more prone to include questions regarding sexual health when taking a patient’s medical history.
